# Statement of Retraction: Neisseria sicca and Corynebacterium matruchotii inhibited oral squamous cell carcinomas by regulating genome stability

**DOI:** 10.1080/21655979.2026.2667622

**Published:** 2026-05-21

We, the Editors and Publisher of the journal *Bioengineered*, have retracted the following article from publication.

Shen, X., Zhang, B., Hu, X., Li, J., Wu, M., Yan, C., Yang, Y., and Li, Y. (2022). Neisseria sicca and Corynebacterium matruchotii inhibited oral squamous cell carcinomas by regulating genome stability. *Bioengineered*, 13(6), 14094–14106. https://doi.org/10.1080/21655979.2022.2078556  

Since publication, significant concerns have been raised about the integrity of the data and reported results in the article.

When approached for an explanation, the authors provided their original data and answered our queries, but these do not sufficiently address the concerns.

As verifying the validity of published work is core to the integrity of the scholarly record, we are therefore retracting the article. The authors have been informed of this decision. The authors do not agree with the retraction.

We have been informed in our decision-making by our policy on publishing ethics and integrity and COPE guidelines. 

The retracted article will remain online to maintain the scholarly record and will be digitally watermarked on each page as ‘Retracted’. 

